# The Metabotropic Purinergic P2Y Receptor Family as Novel Drug Target in Epilepsy

**DOI:** 10.3389/fphar.2018.00193

**Published:** 2018-03-07

**Authors:** Mariana Alves, Edward Beamer, Tobias Engel

**Affiliations:** Department of Physiology & Medical Physics, Royal College of Surgeons in Ireland, Dublin, Ireland

**Keywords:** epilepsy, status epilepticus, pharmacoresistance, purinergic signaling, metabotropic P2Y receptors

## Abstract

Epilepsy encompasses a heterogeneous group of neurological syndromes which are characterized by recurrent seizures affecting over 60 million people worldwide. Current anti-epileptic drugs (AEDs) are mainly designed to target ion channels and/or GABA or glutamate receptors. Despite recent advances in drug development, however, pharmacoresistance in epilepsy remains as high as 30%, suggesting the need for the development of new AEDs with a non-classical mechanism of action. Neuroinflammation is increasingly recognized as one of the key players in seizure generation and in the maintenance of the epileptic phenotype. Consequently, targeting signaling molecules involved in inflammatory processes may represent new avenues to improve treatment in epilepsy. Nucleotides such as adenosine-5′-triphosphate (ATP) and uridine-5′-triphosphate (UTP) are released in the brain into the extracellular space during pathological conditions such as increased neuronal firing or cell death. Once released, these nucleotides bind to and activate specific purinergic receptors termed P2 receptors where they mediate the release of gliotransmitters and drive neuronal hyperexcitation and neuroinflammatory processes. This includes the fast acting ionotropic P2X channels and slower-acting G-protein-coupled P2Y receptors. While the expression and function of P2X receptors has been well-established in experimental models of epilepsy, emerging evidence is now also suggesting a prominent role for the P2Y receptor subfamily in seizure generation and the maintenance of epilepsy. In this review we discuss data supporting a role for the P2Y receptor family in epilepsy and the most recent finding demonstrating their involvement during seizure-induced pathology and in epilepsy.

## Introduction

The primary treatment for epilepsy is the use of anti-epileptic drugs (AEDs). These drugs control seizures by shifting the balance of inhibitory and excitatory drive in the brain (Bialer et al., 2013). 30% of patients, however, are pharmacoresistant to all available AEDs and between 40 and 50% of patients on AEDs suffer adverse effects (Baker et al., 1997). Current major goals of epilepsy research are to develop treatment strategies that impact upon disease emergence and progression, show efficacy within the currently pharmacoresistant cohort and have a lower burden of adverse effects. To this end, the role of neuroinflammation in icto- and epileptogenesis is receiving growing attention (Terrone et al., 2017). Purinergic signaling provides a mechanism by which hyperexcitation can lead to an inflammatory response and whereby inflammation can lead to hyperexcited networks. As such, the targeting of purinergic signaling is a promising strategy for developing new treatment options (Engel et al., 2016). Purinergic signaling is mediated via two families of purinergic receptors: ionotropic P2X receptors and metabotropic P2Y receptors (Burnstock, 2007), both receptor subtypes responding to extracellular adenine or uridine nucleotides. While much of the focus of purinergic signaling in epilepsy has focussed on the P2X receptor family, the role of P2Y receptors in epilepsy has, to date, received much less attention (Engel et al., 2016; Rassendren and Audinat, 2016; Beamer et al., 2017). In this review, we summarize data from the emerging field and suggest directions in which P2Y research in epilepsy should develop.

## Seizures, Status Epilepticus, And Epilepsy

Seizures are a transient symptom resulting from abnormally excessive or synchronous neuronal firing in the brain (Fisher et al., 2014). In general, seizures do not last longer than 1–2 min and are self-limiting (Jenssen et al., 2006). Prolonged or recurrent seizures without intervening recovery periods, however, are classified as status epilepticus, a medical emergency (Betjemann and Lowenstein, 2015). Beyond epilepsy, seizures can have many etiologies, including acute insults, such as fever, hypoxia, low blood sugar, brain tumors, lack of sleep, substance abuse, or traumatic brain injury (TBI). Seizures can be classified according to their etiology, semiology, and anatomical focus (Chang et al., 2017). The transition from seizures to status epilepticus is often due to a failure of endogenous anticonvulsant mechanisms, such as the internalization or desensitization of γ-aminobutyric acid (GABA)_A_ receptors (Naylor and Wasterlain, 2005; Wasterlain et al., 2009; Betjemann and Lowenstein, 2015). Status epilepticus is the second most common neurological emergency behind stroke, with an annual incidence of 10–41 cases per 100,000 (Hesdorffer et al., 1998). It is associated with high mortality (up to 20%), morbidity and considerable costs to the health-care system (Betjemann and Lowenstein, 2015) and can cause severe damage to the brain, leading to serious neurological complications such as cognitive impairment (Korngut et al., 2007), and the development of chronic epilepsy (Hesdorffer et al., 1998).

Where seizures are recurrent and spontaneous, epilepsy is diagnosed. According to the International League Against Epilepsy (ILAE), epilepsy is defined by any of the following conditions: “(1) at least two unprovoked (or reflex) seizures occurring > 24 h apart; (2) one unprovoked (or reflex) seizure and a probability of further seizures similar to the general recurrence risk (at least 60%) after two unprovoked seizures, occurring over the next 10 years; and (3) diagnosis of an epilepsy syndrome” (Fisher et al., 2014). Epilepsy is one of the most common neurological disorders, globally. With an incidence of ∼1%, epilepsy affects over 65 million people worldwide (Moshe et al., 2015). This is associated with a global disease burden of 7M disability adjusted life years (DALYs) (Leonardi and Ustun, 2002) and with an estimated annual cost of over €20 billion in Europe alone according to the World Health Organization (2010). Beside the occurrence of spontaneous seizures, epilepsy is associated with an increased mortality and co-morbidities such as anxiety and depression, which severely impact quality of life (Moshe et al., 2015). Epilepsy affects people of all ages, but is most common in the young and, particularly, the elderly (Everitt and Sander, 1998). Epilepsy can either be innate or acquired, arise due to genetic mutations or via epigenetic mechanisms (Chen et al., 2017), structural or metabolic alterations (Reid and Staba, 2014), infection and immune dysregulation (Vezzani et al., 2011), some combination thereof or, as is often the case, be of unknown etiology (idiopathic epilepsy) (Pal et al., 2016). Common mutations underlying epilepsy include those affecting the function of ion channels, such as the Na^+^ channel, Voltage-Gated Sodium Channel Alpha Subunit (SCN1A) (Kasperaviciute et al., 2013), reducing the action potential threshold in neurons. Structural causes often arise as a result of changes in neuronal network connectivity following an initial insult to the brain, such as head injury, stroke, or status epilepticus (Pitkanen et al., 2015). Epileptogenesis, the process of a normal brain becoming epileptic, is usually the result of a precipitating injury and characterized by an interplay of factors including ongoing cell death, inflammation and synaptic and axonal plasticity changes (Pitkanen et al., 2015). Temporal lobe epilepsy (TLE), the most prevalent form of acquired epilepsy, is characterized by hippocampal sclerosis, including neuronal loss, mossy fiber sprouting and the formation of aberrant neuronal networks which can form a unilateral seizure focus, typically in the CA3 region of the hippocampus (Rao et al., 2006), from which seizures often generalize. Possibly because of the importance of these network changes, TLE is associated with a particularly high prevalence of pharmacoresistance (Zhao et al., 2014).

### Current Treatments for Epilepsy and Status Epilepticus

Over 25 AEDs are currently used in the clinic (Bialer and White, 2010). Despite the relatively large range of options available, where the mechanisms of action are understood, they fit into three broad categories: increasing inhibitory transmission (e.g., the glutamate decarboxylase catalyst, Gabapentin), decreasing excitatory transmission [e.g., the non-competitive alpha-amino-3-hydroxy-5-methyl-4-isoxazoleproprionic acid (AMPA) receptor antagonist, Perampanel] and blockade of voltage-gated ion channels (e.g., Na^+^ channel blocker, lamotrigine) (Bialer and White, 2010). In most cases, AEDs have multiple actions and are incompletely understood. For example, Topiramate exerts an inhibitory effect on Na^+^ conductance, enhances GABA neurotransmission via unknown mechanisms, and antagonizes AMPA receptors (Shank et al., 2000). While there is a superficial diversity in mechanisms, all treatment options rely on the concept of redressing a balance between excitatory and inhibitory drive. This has proven largely successful in controlling seizures, but no treatments have been developed that act on the emergence or progression of the epileptic condition. Further, approximately 30% of patients remain pharmacoresistant to all available AEDs; in most cases leaving surgery as their sole remaining option (Moshe et al., 2015). Choice of treatment strategy is based on seizure type, epilepsy syndrome, health problems, other medication used, lifestyle of the patients and considerations such as pregnancy (Moshe et al., 2015; Kinney and Morrow, 2016). AEDs are the frontline treatment for epilepsy. Although strides have been made in terms of safety, tolerability, and pharmacokinetics with the new generation of AEDs, such as felbamate, gabapentin, lamotrigine or oxcarbazepine, the number of patients resistant to all treatments has not moved from 30% for approximately 80 years (Bialer et al., 2013; Moshe et al., 2015) and the search for mechanisms which could disrupt the emergence or progression of the disease remains elusive. There is therefore an urgent need to identify new drug targets which can show efficacy in patients who are currently refractory to available treatment, and can demonstrate a disease modifying effect.

When treating status epilepticus, time is a key factor and terminating the seizure is the number one priority for preventing lasting damage. A protocol for treatment of status epilepticus has been developed whereby, a first line treatment is administered within 5–10 min of seizure onset, a second line treatment is administered within 20–40 min and a third line treatment around 60 min following seizure onset (Shorvon et al., 2008). The best first line treatment is with benzodiazepines, such as lorazepam, diazepam, or midazolam (Betjemann and Lowenstein, 2015). Evidence supporting the best treatment strategy for second and third line treatments is weaker, however current practice involves the use of AEDs such as fosphenytoin, valproic acid or levetiracetam (Glauser et al., 2016) and anesthetic drugs (Betjemann and Lowenstein, 2015). As with epilepsy, approximately 30% of status epilepticus patients are refractory to available drug treatment and these patients are particularly vulnerable to adverse clinical outcomes (Novy et al., 2010). In summary, the drug development challenges for epilepsy and status epilepticus are similar, with a need in both cases for drugs which show efficacy in currently pharmacoresistant patients, while reducing comorbidities and adverse drug effects. In the case of epilepsy, preventing the emergence or progression of the disorder is also an important goal.

### New Directions in Drug Development for Epilepsy

While drugs targeting excitatory and inhibitory drive have proven widely successful in controlling seizures (Bialer et al., 2013), it seems likely that in order to modify disease progression or offer efficacious drug treatment to currently pharmacoresistant epilepsy patients, alternative targets, with a novel mechanism of action, must be sought. Several experimental and clinical findings have demonstrated an important role for neuroinflammation in both icto- and epileptogenesis (Vezzani et al., 2011, 2016). High levels of inflammatory mediators are present in the brains of both experimental rodent models of epilepsy and epilepsy patients (Aronica et al., 2017) and these processes have therefore received much attention in recent years. Selective blockade of the pro-inflammatory cytokine, Interleukin-1β (IL-1β), has been shown to reduce seizures in *in vivo* models of epilepsy (Ravizza et al., 2008; Vezzani et al., 2009), while in an epileptogenesis-resistant animal, the Amazon rodent, *Proechimys*, no acute brain inflammatory response was found following experimentally-induced status epilepticus (Scorza et al., 2017).

Following an insult, such as a seizure or period of status epilepticus, pro-inflammatory cytokines, such as IL-1β, tumor necrosis factor-α (TNF-α) and IL-6 are released in the brain, primarily from astrocytes and microglia (Terrone et al., 2017). These pro-inflammatory cytokines exert a number of effects that contribute to a reduction in the seizure threshold and emergence of chronic epilepsy. Experimental evidence demonstrates that pro-inflammatory cytokines can have an effect on the firing properties of neurons directly, through the modulation of voltage-gated Na^+^, Ca^2+^, and K^+^ ion channels (Viviani et al., 2007), facilitation of excitatory neurotransmission through both pre- and post-synaptic mechanisms, and disinhibition via antagonism of GABA_A_ receptors (Garcia-Oscos et al., 2012). The effect of inflammation on seizures and epilepsy, however, is not limited to direct modulation of the excitatory/inhibitory balance. Gliosis, gliotransmission, increased permeability of the blood–brain barrier (BBB) and subsequent influx of peripheral cells and modulatory molecules, neuronal cell death and the aberrant reorganization of neuronal networks can all be consequences of a neuroinflammatory response (Vezzani et al., 2012). The causality between hyperexcitation, excitotoxicity, and neuroinflammation is circular and, as described below, intercellular signaling through purines is an important mediator of these processes, making purinergic receptors an attractive treatment target.

## Purinergic Signaling

It was not until 1972 that the role of adenosine-5′-triphosphate (ATP) as an intercellular molecule, was first described by Burnstock (1972). Today, it is well-recognized that a wide variety of nucleotides, including ATP, function as either sole or co-transmitter in both the peripheral and central nervous system (CNS). ATP can act as a fast, excitatory neurotransmitter or as a neuromodulator and is involved in a vast array of short- and long-term physiological and pathological processes including inflammation, cellular survival, proliferation, cellular differentiation, and synaptic plasticity (Burnstock et al., 2011; Khakh and North, 2012; Idzko et al., 2014). It has therefore been implicated in numerous different diseases of the CNS including epilepsy (Burnstock, 2017).

### Purine Release in the Brain

Purines and pyrimidines are a well-established source of energy in all living cells. These molecules, however, also play an important role in intercellular communications within the CNS (Lecca and Ceruti, 2008; Idzko et al., 2014). Adenine and uridine nucleotides are present in almost every synaptic and secretory vesicle where they are either present alone, functioning as a fast neurotransmitter or co-stored with classical neurotransmitters (e.g., GABA or glutamate) (Abbracchio et al., 2009). Under physiological conditions, adenine and uridine nucleotides are usually present at micromolar concentrations in the extracellular space; however, under pathological conditions (e.g., inflammation, hyperexcitability, and cell death) extracellular nucleotide levels can reach the milimolar range (Dale and Frenguelli, 2009; Idzko et al., 2014; Rodrigues et al., 2015). ATP [and most likely uridine-5′-triphosphate (UTP)] can enter the extracellular space by crossing the compromised membranes of damaged and dying cells (Rodrigues et al., 2015). In addition, purines are actively released from different cell types including neurons, astrocytes, microglia, and endothelial cells to act as neuro- and glio-transmitters (Lecca and Ceruti, 2008; Rodrigues et al., 2015). Several mechanisms have been proposed to contribute to the release of nucleotides into the extracellular medium including cell damage, exocytosis of secretory granules, vesicular transport involving the vesicular nucleotide transporter (VNUT) and membrane channels such as ABC transporters, pannexins, connexins and via purinergic receptors themselves (Lecca and Ceruti, 2008; Rodrigues et al., 2015). Once released into the extracellular space, adenine and uridine nucleotides are rapidly metabolized by ectonucleotidases (e.g., ecto-nucleoside triphosphate diphosphohydrolases, ectonucleotide pyrophosphatase, alkaline phosphatases, ecto-5′-nucleotidase, and ecto-nucleoside diphosphokinase) into different breakdown products including adenosine-5′-diphosphate (ADP), adenosine, uridine-5′-diphosphate (UDP), and uridine. These metabolites, in turn, are important neurotransmitters/neuromodulators in their own right, with specific receptors for each expressed throughout the CNS (Zimmermann, 2006; Burnstock, 2007).

Direct evidence for ATP release during seizures is mixed. Large elevations in ATP on electrical stimulation of the cortex (Wu and Phillis, 1978) provided the first direct evidence that high levels of neuronal activity could induce the release of ATP. Subsequently, stimulation of the Schaffer collateral in hippocampal slices was demonstrated to induce ATP release in a Ca^2+^-dependent, but glutamate receptor activation-independent manner (Wieraszko et al., 1989), suggesting the release of ATP was pre-synaptic. While ATP release was not detected following high frequency stimulation or electrically-induced epileptiform seizure like events in hippocampal slices (Lopatar et al., 2015), the induction of epileptiform activity in rat hippocampal slices with the use of the mGluR5-agonist, (S)-3,5-Dihydroxyphenylglycine induced the release of ATP through pannexin hemichannels (Lopatar et al., 2015). ATP release was also elevated in hippocampal slices in a high K^+^ model of seizures (Heinrich et al., 2012). Dona et al. (2016) used microdialysis and high-performance liquid chromatography in order to attempt to measure extracellular concentrations of ATP and its metabolites *in vivo* after pilocarpine-induced status epilepticus and following the onset of chronic epilepsy. They found no change in ATP concentrations for 4 h following status epilepticus, but a marked increase in ATP metabolites, including adenosine monophosphate (AMP) and ADP. Concentrations of ATP and all metabolites were reduced during chronic epilepsy, but ATP was elevated by 300% during spontaneous seizures. Because ectonucleotidases rapidly hydrolyze ATP in the extracellular space and the concentration and activity of these enzymes are increased following seizures (Nicolaidis et al., 2005), it is difficult to measure changes in ATP release directly. Less interest has been shown in investigating UTP release following seizures, however, Koizumi et al. (2007) demonstrated that following kainic acid (KA)-induced-seizure-like events in hippocampal slices, extracellular concentrations of UTP were elevated approximately threefold (Koizumi et al., 2007).

Whereas the anticonvulsive properties of the nucleoside, adenosine, are well-documented (Boison, 2016), the possible contribution of extracellular nucleotides to seizure pathology is a relatively new research area (Engel et al., 2016). The discovery of increased extracellular levels of ATP in seizure-prone rats was one of the first studies to suggest a functional contribution of extracellular nucleotides to seizures (Wieraszko and Seyfried, 1989). Demonstrating a direct impact on seizures, another early study showed that the microinjection of ATP analogs into the prepiriform cortex led to the generation of motor seizures (Knutsen, 1997). More recent evidence implicating extracellular nucleotides in seizure generation stems from studies showing that the injection of ATP into the brain of mice led to the development of high spiking on the electroencephalogram (EEG) and exacerbated seizure severity during status epilepticus (Engel et al., 2012; Sebastian-Serrano et al., 2016). In contrast, treatment with UTP decreases the rate of neuronal firing in epileptic rats (Kovacs et al., 2013) and in mice subjected to status epilepticus (Alves et al., 2017). Further, UTP metabolites such as uridine reduce epileptic seizures in patients with epileptic encephalopathy (Koch et al., 2017).

### P2 Receptor Family

Once released, extracellular adenine and uridine nucleotides bind to and activate specific cell surface receptors termed P2 receptors which are ubiquitously expressed and functional on all cell types in the CNS (Burnstock, 2007). The P2 family of receptors include the ionotropic P2X channels and the metabotropic P2Y receptors. The fast acting P2X channels are a family of seven cation-permeable ionotropic receptor subunits (P2X1-7) which form both homo- and hetero-trimers, depolarizing the cell membrane upon activation (Khakh and North, 2006). All P2X receptors are activated by their main endogenous agonist, ATP, and are permeable to small cations including Na^+^, K^+^, and Ca^2+^. All P2X receptor subunits share a common topology with two transmembrane domains, a large extracellular loop and an intracellular amino and carboxyl terminus (Khakh and North, 2006; Burnstock, 2007). Much attention has been paid to the study of P2X receptors over the past decades, in particular in diseases of the CNS (Burnstock et al., 2011; Saez-Orellana et al., 2015). P2X receptor activation has been implicated in numerous pathological conditions including neurodegeneration, inflammation, ischemia, brain trauma, and hyperexcitability (Engel et al., 2016; Burnstock, 2017). Among the P2X receptor subtypes, the P2X7 receptor has attracted by far the most attention as a potential therapeutic target for brain diseases (Sperlagh and Illes, 2014; Rech et al., 2016).

While the P2X receptor family is made up of fast acting ligand-gated ion channels, the metabotropic P2Y receptor family consists of eight G-protein coupled slower-acting receptors: P2Y_1_, P2Y_2_, P2Y_4_, P2Y_6_, P2Y_11_, P2Y_12_, P2Y_13_, and P2Y_14_ (von Kugelgen, 2006; Burnstock, 2007). In contrast to P2X channels, P2Y receptors can be activated by more than one substrate including the adenine nucleotides ATP (P2Y_2_ and P2Y_11_) and ADP (P2Y_1_, P2Y_12_, and P2Y_13_) and the uridine nucleotides UTP (P2Y_2_ and P2Y_4_), UDP (P2Y_6_ and P2Y_14_), and UDP-glucose (P2Y_14_). P2Y receptors contain the typical features of G-protein-coupled receptors which includes an extracellular amino terminus, intracellular carboxyl terminus and seven transmembrane-spanning motifs (Jacobson et al., 2015). P2Y receptors can be further subdivided into groups based on their coupling to specific G proteins. P2Y_1_, P2Y_2_, P2Y_4_, P2Y_6_, and P2Y_11_ receptors are coupled to Gq proteins, which stimulate phospholipase C, ultimately resulting in the subsequent release of Ca^2+^ from intracellular stores and activation of protein kinase C (PKC). Of these, P2Y_11_ receptor can also couple to Gs, stimulating adenylate cyclase and increasing the production of cyclic adenosine monophosphate (cAMP) (von Kugelgen, 2006). P2Y_12_, P2Y_13_, and P2Y_14_ are coupled to Gi proteins, inhibiting adenylate cyclase and thereby decreasing cAMP production (von Kugelgen, 2006).

### Involvement of P2Y Receptor Signaling in Brain Inflammation and Excitability

P2Y receptors are involved in a myriad of different cellular functions and pathological processes pertinent to the process of epileptogenesis and epilepsy including neuroinflammation, neurodegeneration, synaptic reorganization, and changes in neurotransmitter release (von Kugelgen, 2006; Jacobson and Boeynaems, 2010; Pitkanen et al., 2015; Guzman and Gerevich, 2016) making them an attractive antiepileptic therapeutic target.

Inflammatory processes in the brain have received much attention over recent years and are thought to play a major role in seizure-induced pathology and the development of epilepsy (Vezzani et al., 2011). The principle ligands for P2Y receptors are the purine, ATP, the pyrimidine, UTP, and their metabolites, such as ADP and UDP (Burnstock, 2007). The role of each receptor in neuroinflammation is dictated by its affinity for different ligands and downstream targets. ATP is both released as a result of inflammation and promotes pro-inflammatory mechanisms. This circular causality can underpin a positive feedback loop whereby neuroinflammation becomes self-sustaining (Idzko et al., 2014). Less is known about the role of UTP in mediating neuroinflammation. The role of different P2Y receptors in mediating neuroinflammation and cell death seems to be divergent (Forster and Reiser, 2015), depending on downstream signaling pathways and mutually antagonistic actions, but is incompletely understood. The P2Y_1_ receptor, activated by the ATP metabolite ADP, is expressed also on astrocytes and activated under conditions of oxidative stress, prompting the release of IL-6 (Fujita et al., 2009). IL-6 has been shown to play an anti-inflammatory role during ‘classic signaling’ involving the binding of IL-6 to the membrane-bound IL-6 receptor which induces the dimerization of the β-receptor glycoprotein 130 (gp130). In contrast however, IL-6 is also critical for pro-inflammatory signaling in a process termed ‘trans-signaling,’ whereby IL-6 stimulates distant cells which only express gp130 in the absence of the IL-6 receptor (Rothaug et al., 2016). A more recent study has shown that in a chronic model of epilepsy, astrocytes from kindled rats show enhanced Ca^2+^-dependent signaling and astroglial hyperexcitability, which requires the activation of the P2Y_1_ receptor (Alvarez-Ferradas et al., 2015). P2Y_1_ antagonism prevented cognitive deficits and neuronal damage in a model of ischemia in mice (Carmo et al., 2014). A recent study also showed improved histological and cognitive outcomes in a model of TBI in mice provided by P2Y_1_ receptor antagonism (Choo et al., 2013). Activation of astrocytic P2Y_2_ receptors promotes astrocyte activation and migration via an interaction with αV-integrin (Wang et al., 2005). The P2Y_2_ receptor has also been shown to play a protective role against chronic inflammation-induced neurodegeneration in a model of Alzheimer’s disease (Kong et al., 2009). A role for the uridine-sensitive P2Y_4_ receptor in mediating neuroinflammation has not been established (Beamer et al., 2016), with progress hamstrung by a lack of specific tools for targeting this receptor. The P2Y_6_ receptor promotes the activation of microglia and the adoption of a phagocytic phenotype following activation by the UTP metabolite UDP (Koizumi et al., 2007). This is dependent on downstream signaling involving phospholipase C and PKC. Other studies have suggested a role for the P2Y_12_ receptor in microglial activation (Ohsawa et al., 2010), showing that activation of integrin-β1 in microglia through P2Y_12_ is involved in directional process extension by microglia in brain tissue. As discussed in more detail below, P2Y_12_-dependent process extension has been shown to be increased following status epilepticus in mice (Eyo et al., 2014).

The effects of P2Y signaling are not limited to inflammatory processes and cellular survival alone. P2Y signaling also impacts directly on neuronal excitability, synaptic strength, and synaptic plasticity (Guzman and Gerevich, 2016). Presynaptic P2Y receptors have been shown to affect the release of different neurotransmitters including glutamate, noradrenaline and GABA, most likely by reducing presynaptic Ca^2+^ influx (Fischer et al., 2009). P2Y_1_, P2Y_2_, and P2Y_4_ inhibit the release of glutamate in the hippocampus (Mendoza-Fernandez et al., 2000; Koizumi et al., 2003; Rodrigues et al., 2005), possibly through the inhibition of voltage-activated Ca^2+^ channels (VACCs) (Gerevich et al., 2004). Using the same mechanism, the release of noradrenaline in the hippocampus was also blocked via P2Y_1_, P2Y_12_, and P2Y_13_ activation (Csolle et al., 2008). Similarly, activation of P2Y_4_ with UTP blocks the release of the inhibitory neurotransmitter GABA from cerebellar basket cells (Donato et al., 2008). P2Y receptors alter the expression/function of other membrane receptors and voltage-gated ion channels. P2Y_1_ triggers the desensitization or internalization of the metabotropic glutamate receptor 1 (mGluR1) (Mundell et al., 2004) and inhibits *N*-methyl-D-aspartate (NMDA) receptor channels (Luthardt et al., 2003). P2Y_1_ also increases the sensitivity of the GABA_A_ receptor (Saitow et al., 2005) and inhibits P2X receptors (Gerevich et al., 2007). P2Y receptor activation can lead to the inhibition of VACCs (Diverse-Pierluissi et al., 1991) thereby potentially influencing neuronal excitability and synaptic plasticity. P2Y receptors also block potassium channels [e.g., voltage-gated potassium channel subunit KvLQT2,3 (Filippov et al., 2006) or G protein-coupled inward rectifying channels 1, 2, and 4 (GIRK1,2,&4)] (Filippov et al., 2004), inhibiting membrane hyperpolarisation and thereby facilitating an increased frequency of neuronal firing (Brown and Passmore, 2009; Guzman and Gerevich, 2016). On a network level, P2Y_1_ increases the firing of GABAergic inhibitory neurons either directly or via P2Y_1_-dependent activation of astrocytes in the hippocampus, eventually leading to an increase in inhibitory-postsynaptic currents (IPSCs) in pyramidal neurons (Bowser and Khakh, 2004). In a more recent study, Jacob et al. (2014) showed that astrocytic P2Y_1_ activation increases extracellular concentrations of GABA by inhibiting Ca^2+^ signaling dependent GABA transport (Jacob et al., 2014). In conclusion, while P2X receptors excerpt a mainly facilitatory effect on synaptic transmission (Khakh and North, 2012), the effects of P2Y receptors seem to be context-specific, either increasing or decreasing neuronal firing by altering excitatory and inhibitory neurotransmitter release or altering receptor function (e.g., NMDA and GABA_A_) and channel conductance (e.g., voltage-gated KCNQ2/3 potassium channel) (Guzman and Gerevich, 2016).

## Purinergic Signaling as a Novel Drug Target in Epilepsy

Mounting evidence has accumulated over the past decades demonstrating a causal role for purinergic signaling in numerous pathological conditions ranging from cancer (Di Virgilio, 2012), cardiovascular disease (Ralevic, 2015), blood cell diseases (McGovern and Mazzone, 2014) to diabetes (Fotino et al., 2015) and brain diseases (Puchalowicz et al., 2014). Among brain diseases, intervention in purinergic signaling has been postulated as a new therapeutic avenue for acute insults to the brain such as stroke (Kuan et al., 2015) and TBI (Kimbler et al., 2012) and for chronic brain diseases including neurodegenerative diseases (e.g., Huntington’s, Alzheimer’s, and Parkinson’s disease) (Miras-Portugal et al., 2016), neuropsychiatric disorders (e.g., depression and schizophrenia) (Burnstock et al., 2011) and also epilepsy (Beamer et al., 2017). Emphasizing the potential for targeting purinergic signaling as a promising new therapeutic strategy, several compounds are already used in the clinic, including the P2Y_2_ agonist Diquafosol for the treatment of dry eye (Lau et al., 2014) or Clopidogrel, a P2Y_12_ antagonist used for the treatment of thrombosis (Sarafoff et al., 2012) while others have progressed into clinical trials such as antagonists of the ionotropic P2X3 used against refractory chronic cough (Abdulqawi et al., 2015) and P2X7 receptors used against rheumatoid arthritis (Keystone et al., 2012) and other inflammatory conditions (Rech et al., 2016).

To date, most of the studies performed to elucidate the changes in expression and functional contribution of purinergic P2 receptors to seizures and epilepsy have focused on the P2X receptor subtype, in particular the P2X7 receptor (reviewed in Beamer et al., 2017), with relatively little attention paid to the P2Y receptor family. The lack of apparent interest was largely due to a lack of suitable tools (e.g., drugs to manipulate P2Y function) and the strong focus on fast synaptic effects conferred by the ionotropic P2X receptors (Engel et al., 2016). Recent studies using experimental animal models of status epilepticus and epilepsy and analysis of patient brain tissue, however, suggest a prominent role for P2Y signaling during seizures and the development of epilepsy (**Table 1**). In the last section of this review we describe in detail the evidence linking a pathological activation of the metabotropic P2Y receptors to seizure generation and seizure-induced pathology and discuss the antiepileptic potential of drugs targeting P2Y signaling.

**Table 1:** Summary of the main findings of P2Y receptor expression and function during status epilepticus and epilepsy in experimental models of epilepsy and patient brain.

Disease process/stage	Epilepsy models/patients	Brain region	Techniques/drugs	Main results	Reference
Status epilepticus	i.p. KA-induced status epilepticus in mice	Hippocampus	GFP reporter mice; hippocampal slices; qPCR; UDP (broad-spectrum P2Y receptor agonist) and 2-MeSADP treatment (P2Y_1_, P2Y_12_, P2Y_13_ agonist)	Upregulation of *P2ry_6_*, *P2ry_12_* transcripts after status epilepticus; transient downregulation of the *P2ry_13_* transcript 3 h following status epilepticus and increased *P2ry_13_* transcript 48 h post-status epilepticus; increased microglia currents after treatment with UDP and 2-MeSADP	Avignone et al., 2008
Status epilepticus	i.p. and i.c.v. KA-induced status epilepticus in mice	Hippocampus	P2Y_12_ knock-out mice	Increased seizure phenotype; reduced hippocampal microglial processes	Eyo et al., 2014
Status epilepticus	i.p. KA-induced status epilepticus in mice	Hippocampus	GFP reporter mice; hippocampal slices and two photon microscopy; 2-MeSADP treatment (P2Y_1_, P2Y_12_, P2Y_13_ agonist)	Increased velocity of microglia process extension toward a pipette containing 2-MeSADP following induction of status epilepticus	Avignone et al., 2015
Status epilepticus	i.p. pilocarpine-induced status epilepticus in mice	Hippocampus	IH	P2Y_1_ activated in neuronal progenitor cells following status epilepticus	Rozmer et al., 2016
Status epilepticus and epilepsy	i.a. KA-induced epilepsy in mice; i.p. pilocarpine-induced statusepilepticus in mice	Hippocampus (mice and patients)	WB; qPCR; i.c.v. treatment with ADP and UTP (broad-spectrum P2Y receptor agonists)	Status epilepticus: Increased *P2ry_2_*, *P2ry_4_*, and *P2ry_6_* and decreased *P2ry_1_*, *P2ry_12_*, *P2ry_13_*, and *P2ry_14_* transcript levels; increased P2Y_1_, P2Y_2_, P2Y_4_, and P2Y_6_ and decreased P2Y_12_ protein levels.	Alves et al., 2017
				ADP exacerbates seizure severity; UTP decreases seizure severity and neuronal death	
	TLE patient brain			Epilepsy: Increased *P2ry_1_*, *P2ry_2_*, and *P2ry_6_* transcripts in mice; increased P2Y_1_, P2Y_2_, and P2Y_12_ protein levels in mice. Increased P2Y_1_ and P2Y_2_ and decreased P2Y_13_ protein levels in TLE patients	
Epilepsy	Patients with intractable epilepsy associated with focal cortical dysplasia	Cortex	WB; IH	Increased P2Y_1_, P2Y_2_, and P2Y_4_ expression in astrocytes	Sukigara et al., 2014
Epilepsy	Rapid kindling protocol in rats	Hippocampus	Hippocampal slices; treatment with P2Y_1_ antagonist MRS2179	Enhanced spontaneous Ca^2+^-dependent signaling and astroglial hyperexcitability via P2Y_1_ antagonism	Alvarez-Ferradas et al., 2015

*GFP, green fluorescent protein; i.a., intraamygdala; i.c.v., intra-cerebro-ventricular; IH, immunohistochemistry; i.p., intraperitoneal; qPCR, quantitative polymerase chain reaction; TLE, temporal lobe epilepsy; WB, western blot*.

### P2Y Expression Following Status Epilepticus

One of the earliest studies analyzing P2Y expression changes following status epilepticus used the intraperitoneal KA-induced status epilepticus mouse model (Avignone et al., 2008). Here, the authors observed an increase in transcription of *P2ry_6_*, *P2ry_12_*, and *P2ry_13_* in the hippocampus. In another study using the intraperitoneal pilocarpine mouse model, Rozmer et al. (2016) show an increase in P2Y_1_ activity in neuronal progenitor cells following status epilepticus. In a more recent study, our group published a comprehensive analysis of changes in transcription and expression across the entire P2Y family of receptors following status epilepticus using two different mouse models: the intraamygdala KA mouse model of status epilepticus (Mouri et al., 2008) and the intraperitoneal pilocarpine mouse model of status epilepticus (Alves et al., 2017). Both, intraamygdala KA and intraperitoneal pilocarpine-induced status epilepticus increased the transcription of the uridine-sensitive P2Y receptors *P2ry_2_*, *P2ry_4_*, and *P2ry_6_* in the hippocampus. At the same time, the transcription of the adenine-sensitive receptors *P2ry_1_*, *P2ry_12_*, and *P2ry_13_* was downregulated. At the protein level, hippocampal levels of P2Y_1_, P2Y_2_, P2Y_4_, and P2Y_6_ were increased and P2Y_12_ was decreased following status epilepticus. No immunohistochemistry was performed to identify cell types expressing the different P2Y receptors. Thus, these results show that changes in the transcription of P2Y receptors following status epilepticus closely correlate with the known profile of agonists (i.e., adenine-sensitive receptors are downregulated and uridine-sensitive receptors are upregulated) and, at the protein level, the G-protein coupling of the receptors with P2Y receptors coupled to Gq being increased and P2Y receptors coupled to Gi being downregulated or not changed (Alves et al., 2017).

### P2Y Expression During Chronic Epilepsy

Much less is known about the expression profile of P2Y receptors during epilepsy. To date, the only study carried out characterizing P2Y expression in experimental epilepsy was undertaken using the intraamygdala KA mouse model (Alves et al., 2017). In this model, mice become epileptic after a short latent period of 2–5 days (Mouri et al., 2008). Analysis of the hippocampus 14 days-post status epilepticus revealed increased *P2ry_1_*, *P2ry_2_*, and *P2ry_6_* transcription and increased P2Y_1_, P2Y_2_, and P2Y_12_ protein levels. No changes were observed for the remaining receptors. Thus, P2Y upregulation seems to be the predominant response during experimental epilepsy, probably due to an increase in inflammatory processes in the epileptic brain. In the same study, resected hippocampal samples from drug-refractory epilepsy patients were also analyzed. In these samples, as seen before in hippocampal samples from epileptic mice, the predominant response was an upregulation of P2Y receptors with P2Y_1_ and P2Y_2_ significantly upregulated. Of note, the only exception, and in contrast to findings from the mouse model of epilepsy, expression of the P2Y_13_ receptor was found at lower levels in the epileptic brain compared to controls (Alves et al., 2017). In another previous study using brain tissue from patients suffering from intractable epilepsy associated with focal cortical dysplasia, Sukigara et al. (2014) showed increased levels of P2Y_1_, P2Y_2_, and P2Y_4_. Interestingly, the authors reported the main increase to be in astrocytes (Sukigara et al., 2014). Thus, P2Y receptor expression is altered during epilepsy, however, in contrast to status epilepticus, the main response was an upregulation of the P2Y receptor family.

### P2Y Function During Status Epilepticus

**FIGURE 1:**
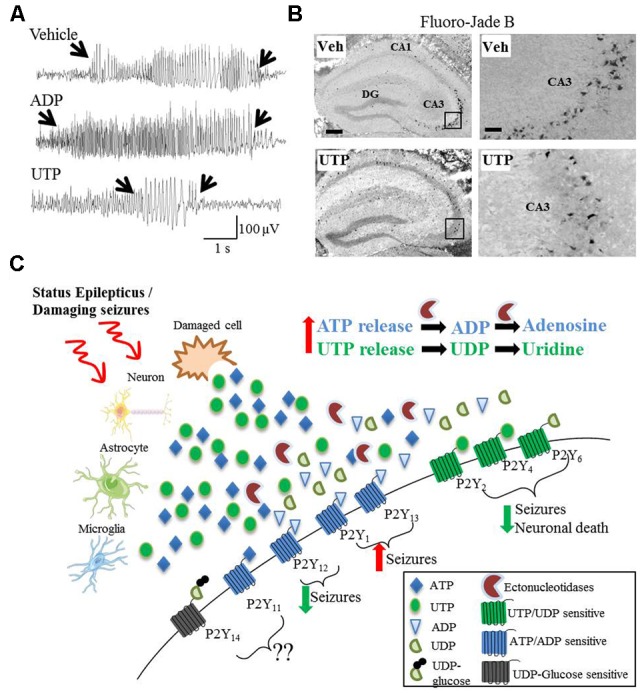
Divergent effects on seizures and seizure-induced pathology of different P2Y agonists. **(A)** Representative EEG traces recorded during intraamygdala KA-induced status epilepticus showing pro-convulsive effects of ADP treatment, while UTP acts as potent anti-convulsive. **(B)** UTP protects against neuronal cell death following status epilepticus, visualized with the neuronal-cell death marker Fluoro-Jade B. **(C)** Hypothetical model of P2Y receptor function during and after status epilepticus or damaging seizures. During and following status epilepticus, purines (e.g., ATP and UTP) are actively released from different cell types including neurons, astrocytes and microglia or enter the extracellular space from damaged/necrotic cells. While the activation of UTP/UDP-sensitive P2Y receptors (P2Y_2_, P2Y_4_, and P2Y_6_) may has anticonvulsive and neuroprotective effects, the activation of ATP/ADP-sensitive P2Y receptors (P2Y_1_ and P2Y_13_) may be pro-convulsive. The role of P2Y_11_ and P2Y_14_ have not been studied so far and therefore are unknown. Counterintuitively, P2Y_12_ is ATP/ADP-sensitive, but seems to be anticonvulsive and neuroprotective.

Despite the involvement of P2Y signaling in numerous pathological processes believed to play a key role during epilepsy, a possible involvement of the different P2Y receptor subtypes to seizure-induced pathology remains poorly explored and only three recent studies have suggested a functional contribution of P2Y receptors to seizures or seizure-induced pathology. The first study demonstrating a causal role for P2Y signaling during status epilepticus used mice deficient in P2Y_12_ (Eyo et al., 2014). P2Y_12_ is one of the most important therapeutic targets of the P2Y receptor family, with P2Y_12_ agonists already routinely used in the clinic as an antithrombotic agent (Cattaneo, 2015). Eyo et al. (2014) report a P2Y_12_-dependent extension of microglial process toward neurons following KA-induced status epilepticus. Neuronal NMDA receptor activation led to an influx of Ca^2+^, stimulating ATP release, which subsequently activated microglial P2Y_12_ receptors, which, in turn stimulated the extension of the processes. Interestingly, P2Y_12_ knock-out mice, in which this process was inhibited, showed an increased seizure severity (Eyo et al., 2014). Thus, the authors concluded that microglial P2Y_12_ receptors are necessary for microglia-neuron interaction during status epilepticus and that microglial process extension via P2Y_12_ may serve an anti-ictal function. In a later study, Avignone et al. (2015) demonstrate that microglial processes extend toward a pipette containing methylthio-ADP, an agonist for P2Y_1_, P2Y_12_, and P2Y_13_ (and a weak agonist for P2Y_11_). The velocity of this chemotaxis was increased in activated microglia following status epilepticus. Because they also found an upregulation of P2Y_12_ in activated microglia, the authors attributed this receptor as the likely mediator of this response (Avignone et al., 2015). More recently, our group has shown seizure altering properties of the broad-spectrum P2Y agonists ADP and UTP in the intraamygdala KA mouse model (Alves et al., 2017). Once status epilepticus was established, mice treated with ADP showed an increased seizure severity and mice treated with UTP showed a strong reduction in seizure severity and accompanying seizure-induced cell death (Alves et al., 2017). These results are in line with protective cellular mechanisms acting during status epilepticus regarding the P2Y receptor family with adenine-sensitive receptors being generally downregulated during status epilepticus and uridine-sensitive receptors being upregulated (**Figure 1**).

In conclusion, while these results demonstrate a causal role for P2Y signaling during status epilepticus, we are still far from a clear and comprehensive picture of how individual P2Y receptors impact on seizure pathology.

### P2Y Function During Chronic Epilepsy

Although results from functional studies during status epilepticus and changes in expression of P2Y receptors during epilepsy strongly suggest a role for these receptors in epilepsy, to date, no studies have been performed to determine the functional contribution of P2Y receptors to epileptogenesis or the epileptic phenotype. Possible reasons are the lack of centrally available P2Y-targeting drugs and the lack of mouse models with conditional deletion of P2Y receptors, both essential for the study of the involvement of P2Y receptors during epilepsy.

## Conclusion and Future Perspectives

What remains to be done to establish P2Y receptors as potential drug target for epilepsy in the future? Despite the exciting emerging data revealing P2Y signaling in the brain, we are only at the beginning of understanding the potential role in seizure generation and during epileptogenesis. Recent studies have shown distinct changes in expression of the P2Y receptor family following status epilepticus and during seizures and a functional contribution has been postulated using broad-spectrum P2Y agonists (ADP and UTP) (Alves et al., 2017) and *P2Y_12_* knock-out mice (Eyo et al., 2014), there are many key issues, however, which will have to be resolved before considering P2Y receptors as valid drug target.

(i) Studies have demonstrated altered P2Y receptor expression following status epilepticus and during epilepsy (Alves et al., 2017). To get a better picture about the potential role of P2Y signaling during seizure-related pathologies, however, we must determine what cell types (e.g., neurons vs. glia; inhibitory vs. excitatory neurons) express the receptor and their sub-cellular localization (e.g., somatic vs. synaptic). (ii) Treatment of mice during status epilepticus with P2Y broad-spectrum agonists suggest a role of these receptors in seizure generation and seizure-induced pathology (Alves et al., 2017), however, we still do not know the role of individual P2Y receptors during seizures, with the only exception being the P2Y_12_ receptor (Eyo et al., 2014). P2Y receptor-specific, centrally available drugs or P2Y knock-out mice, if possible cell-specific, must be used to determine the possible impact of the different P2Y receptors on seizures and epilepsy. (iii) P2Y receptors have been shown to be involved in numerous pathological processes in the brain (Beamer et al., 2016), however, signaling downstream of P2Y during seizures and epilepsy remains elusive, with the only exception being P2Y_12_ functioning on microglia (Eyo et al., 2014). P2Y receptors have been shown to alter both excitatory (e.g., glutamate) and inhibitory neurotransmitter release in the brain (Garcia-Oscos et al., 2012), therefore, future studies must determine whether P2Y signaling impacts on the release of neurotransmitters and what neurotransmitters are altered during seizures. Do seizure-induced changes in P2Y function impact on the function of other cell membrane channels/receptors (e.g., potassium channels, calcium channels, NMDA receptors, GABA receptors) thereby altering neuronal excitability? (iv) Different P2Y receptors respond to different agonists (e.g., UTP, UDP, ATP, and ADP) (von Kugelgen, 2006), however, we still do not know at what concentrations these nucleotides are available during seizures/epilepsy and when, where and from which cell types these nucleotides are released or what mechanisms (e.g., ectonucleotidases) are responsible for extracellular nucleotide concentration changes. (v) To date, studies have solely used the KA and pilocarpine mouse model of status epilepticus to analyze P2Y signaling during seizures (Avignone et al., 2008, 2015; Eyo et al., 2014; Alves et al., 2017). These mouse models rely, however, on chemically-induced seizures and only recapitulate certain aspects of the disease (Reddy and Kuruba, 2013). Results must therefore be confirmed in other models of acute seizures and chronic epilepsy. (vi) To date, we do not know what drives P2Y receptor expression during seizures. The clear expression pattern according to P2Y receptor agonists during status epilepticus, however, points toward common pathways. The identification of what drives P2Y expression during and following seizures may also therefore provide much needed new target genes for seizure control. (vii) While changes in P2Y receptor expression and, to an extent, function, have been analyzed in hippocampal tissue, extrahippocampal brain areas, in particular the cortex, may also contribute to the epilepsy phenotype (Thompson and Duncan, 2005; Helmstaedter, 2007). Status epilepticus is associated with significant extrahippocampal injury, including in the cortex (Fujikawa et al., 2000) and cortical thinning has also been reported in patients with pharmacoresistant TLE (Bernhardt et al., 2010). Consequently, the P2Y expression profile must also be analyzed in non-hippocampal brain regions. (viii) Data obtained by using the broad-spectrum agonists ADP and UTP with ADP exacerbating and UTP decreasing seizure pathology (Alves et al., 2017), suggest that a mix of antagonist (e.g., adenine-specific receptors) and agonists (e.g., uridine-specific receptors) may provide better protection than single receptor targeting.

In conclusion, P2Y signaling is altered during and after status epilepticus and during epilepsy. Functional studies demonstrate an involvement of P2Y receptors in seizure pathology. Despite promising results, however, we are only at the beginning of understanding the role of P2Y signaling during seizures to ultimately establish P2Y targeting as possible therapeutic avenue in epilepsy.

## Author Contributions

MA wrote the manuscript and designed the Figure and Table. EB edited the manuscript. TE wrote and edited the manuscript.

## Conflict of Interest Statement

The authors declare that the research was conducted in the absence of any commercial or financial relationships that could be construed as a potential conflict of interest.
